# Cathepsin K inhibition induces Raptor destabilization and mitochondrial dysfunction via Syk/SHP2/Src/OTUB1 axis-mediated signaling

**DOI:** 10.1038/s41419-023-05884-z

**Published:** 2023-06-17

**Authors:** Seung Un Seo, Seon Min Woo, Taeg Kyu Kwon

**Affiliations:** 1 Department of Immunology, School of Medicine, Keimyung University, Daegu, 42601 South Korea; 2 Center for Forensic Pharmaceutical Science, Keimyung University, Daegu, 42601 South Korea

**Keywords:** Chemotherapy, Apoptosis

## Abstract

The Raptor signaling pathway is a critical point of intervention in the invasion and progression of cancer. The non-receptor tyrosine kinase Src-mediated phosphorylation of OTUB1-Y26 plays a critical role in Raptor stabilization, whereas cathepsin K inhibitor (odanacatib; ODN) and knockdown (siRNA) induce Raptor destabilization. However, the mechanisms involved in cathepsin K inhibition-induced OTUB1-Y26 phosphorylation in Raptor stabilization have not been yet elucidated. This study showed that cathepsin K inhibition activates SHP2, a tyrosine phosphatase, that dephosphorylates OTUB1 and destabilizes Raptor, whereas SHP2 deletion and pharmacological inhibition increase OTUB1-Y26 phosphorylation and Raptor expression. SHP2 deletion also led to the inhibition of ODN-induced mitochondrial ROS, fusion, and dysfunction. Furthermore, cathepsin K inhibition phosphorylated spleen tyrosine kinase (Syk) at Y525 and Y526, resulting in the SHP2-mediated dephosphorylation of OTUB1-Y26. Collectively, our findings identified Syk not only as an upstream tyrosine kinase required for SHP2 activation but also showed a critical mechanism that regulates ODN-induced Raptor downregulation and mitochondrial dysfunction. In conclusion, Syk/SHP2/Src/OTUB1 axis-mediated signaling can act as a therapeutic target in cancer management.

## Introduction

Cathepsin K is a papain-like cysteine protease that degrades several components of the extracellular matrix, thus playing a crucial role in cancer invasion and progression [1–3]. Cathepsin K is a well-known therapeutic target in osteoclasts; however, its expression increases in cancer cells and is related to cancer metastasis [4–8]. Moreover, the shRNA-mediated knockdown of cathepsin K inhibits the proliferation and metastasis of breast cancer cells [9]. In preclinical studies, several cathepsin K inhibitors such as relacatib (SB-462795), L-235, balicatib (AAE-581), ONO-5334, and odanacatib (ODN) have been reported [6]. Above all, ODN, a reversible cathepsin K inhibitor, is in a phase III clinical trial for osteoporosis treatment [10]. Clinical trial of ODN in women with breast cancer and established bone metastases revealed the suppression effect of bone resorption [11].

Most recently, our previous study reported that targeting cathepsin K using siRNA or ODN enhances cancer cells’ sensitivity to sub-lethal doses of anti-cancer drugs by triggering pro-apoptotic protein Bim stabilization [12]. In addition, cathepsin K inhibition also increases Bax mRNA and protein expression via OTUB1-mediated p53 stabilization [13]. Further study demonstrated that ODN-mediated Bax upregulation is due to the stabilization of the Sp1 transcription factor, regardless of p53 [14]. ODN-mediated upregulation of Bim and Bax is caused by the degradation of Raptor protein and mitochondrial dysfunction [12–14]. Src-mediated OTUB1 phosphorylation at tyrosine 26 residue (Y26) plays an important role in Raptor stabilization [15]. Collectively, these results suggested that the major determinant of sensitization of anti-cancer drugs by ODN is caused by Raptor destabilization-mediated mitochondrial dysfunction.

In this study, we investigated the molecular mechanisms involved in Raptor destabilization during cathepsin K inhibition. In ODN-treated cells, Syk, a tyrosine kinase, activates SHP2 resulting in the induction of dephosphorylation of Src and OTUB1. Thus, in cathepsin K inhibition-mediated Raptor degradation, Syk activation appears upstream affecting the activation of downstream target proteins and eventually regulating the entire pathway. Our results suggest that Syk/SHP2/Src/OTUB1 signaling axis is critical in cathepsin K inhibition-mediated Raptor degradation and mitochondrial dysfunction.

## Materials and methods

### Cells and materials

All human cancer cells used in this study (Caki-1, A549, and DU145) were obtained from the American Type Culture Collection (Manassas, VA, USA). The cells were cultured in Dulbecco’s modified Eagle’s medium containing 10% fetal bovine serum (Welgene, Gyeongsan, Korea), 1% penicillin–streptomycin, and 100 µg/mL gentamycin (Thermo Fisher Scientific, Waltham, MA). Information on the materials used is provided in Supplementary Table 1.

### Knockdown using siRNA

The GFP (control) and cathepsin K siRNAs were obtained from Santa Cruz Biotechnology (St. Louis, MO, USA). The tyrosine phosphatases (SHS-0190-2) and tyrosine kinases (SHS-0110-7) siRNAs Subset were obtained from Bioneer (Daejeon, Korea). Cancer cells were transfected using Lipofectamine RNAiMAX (Thermo Fisher Scientific, Waltham, MA, USA).

### Western blotting

Cells were lysed using RIPA buffer containing protease inhibitors [16], and centrifuged at 13,000 × *g* at 4 °C for 15 min to obtain proteins. Proteins were then separated on SDS-PAGE and transferred onto nitrocellulose membranes (GE Healthcare Life Science, Pittsburgh, PA). Protein expression was detected using an enhanced chemiluminescence kit (EMD Millipore). Information regarding the antibodies used is listed in Supplementary Table 2.

### Measurement of mitochondrial ROS

Mitochondrial ROS generation was analyzed using a 2 μM MitoSOX Red mitochondrial superoxide indicator (Thermo Fisher Scientific, Waltham, MA, USA). Cells were stained with 2 μM MitoSOX Red dye for 10 min before trypsinization. Stained cells were resuspended in PBS and mitochondrial ROS production was measured using a BD Accuri^TM^ C6 cytometer (BD Biosciences, San Jose, CA, USA) and a fluorescence microscope (Carl Zeiss, Jena, Germany).

### Detection of mitochondrial mass

MitoTracker Deep Red stains active mitochondria to show mitochondrial respiration, whereas MitoTracker Green dye stains all mitochondria to represent the mitochondrial mass [17]. To assess mitochondrial damage, Caki-1 cells were stained with 1 μM MitoTracker Deep Red and 1 μM MitoTracker Green dye (Molecular Probes Inc., Eugene, OR, USA) for 15 min at 37 °C. After trypsinization, cells were washed in PBS, and mitochondrial damage was assessed using a FACSCanto^TM^ flow cytometer (BD Biosciences, San Jose, CA, USA).

### Evaluation of mitochondrial dynamics

Mitochondrial morphology in Caki-1/pDsRed2-Mito cells was observed using a fluorescence microscope (Carl Zeiss, Jena, Germany). Mitochondrial length (>10 μm considered as elongated and <10 μm considered as fragmented) was quantified using ZEN3.4. The average length of at least 20 mitochondria was measured for each dataset, in three independent experiments.

### Flow cytometry analysis

Trypsinized cells were fixed using 95% ethanol at 4 °C. After 1 h, the cells were resuspended in 1.12% sodium citrate buffer (pH 8.4) containing 12.5 µg of RNase, and incubated at 37 °C for 30 min. After incubation, 250 µL of PI (50 µg/mL) was added to the cells and further incubated at 37 °C for 30 min. For apoptosis analysis, the sub-G1 population was measured using a BD Accuri^TM^ C6 flow cytometer (BD Biosciences, San Jose, CA).

### In vivo xenograft model

Male BALB (bagg and albino)/c-nude mice were subcutaneously injected with cancer cells into the flank of each mouse. After 3 weeks, mice were intraperitoneally injected with 5 mg/kg ODN (in 2% DMSO/PBS) or 5 mg/kg oxaliplatin (PBS)] for 20 days, and the modulation of protein expression was identified using the same sample used previously [13].

### Survival rate of patients with renal clear carcinoma (RCC)

The overall survival of patients, according to Syk, SHP2, and Src expression, was obtained from Gepia2 (http://gepia2.cancer-pku.cn/) from The Cancer Gene Atlas (TCGA) cohort with high versus low expression [18].

### Patient specimens

In total, 40 renal cell carcinoma specimens and adjacent normal tissues were collected from the Keimyung University Dongsan Hospital Biobank (IRB-2019-11-040).

### Statistical analysis

Statistical analyses were performed using the Statistical Package for Social Sciences (SPSS, Version 26.0; IBM SPSS, Armonk, NY, USA). All experiments were repeated in triplicates. Data are presented as mean ± SD and were analyzed using one-way ANOVA and post hoc comparisons (Student–Newman–Keuls test).

## Results

### Cathepsin K inhibition induces Raptor destabilization through dephosphorylation of OTUB1 at Y26

**Fig. 1:**
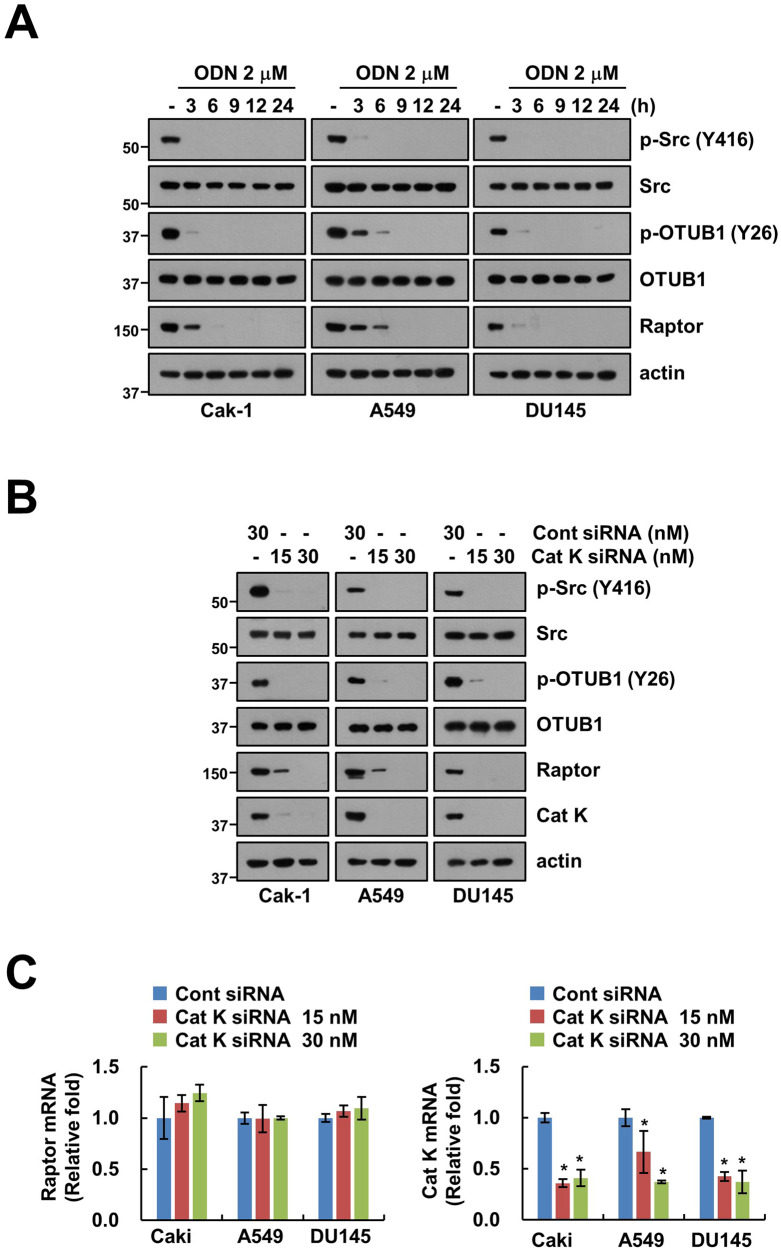
Inhibition of cathepsin K dephosphorylates Src and OTUB1 resulting in downregulation of Raptor. **A** Cancer cells (Caki-1, A549, and DU145) were treated with 2 μM ODN for the indicated periods. **B**, **C** Cancer cells (Caki-1, A549, and DU145) were transfected with control (Cont) siRNA or Cat K siRNA for 24 h. Protein (**B**) and mRNA (**C**) expression were determined using western blotting and qPCR, respectively.

We previously reported that Src tyrosine kinase phosphorylates OTUB1 at Y26 residue and plays a critical role in Raptor stability [15]. Contrastingly, cathepsin K inhibition in cancer cells caused the destabilization of Raptor [12]. To investigate the involvement of OTUB1 and Src in cathepsin K inhibitor (odanacatib; ODN)-mediated Raptor destabilization, we determined the phosphorylation of OTUB1 and Src in ODN-treated cells using a specific phospho-antibody. Results indicated that ODN treatment induces OTUB1 (Y26) and Src (Y416) dephosphorylation in a time-dependent manner (Fig. 1A). Similar results were also observed in siRNA-mediated knockdown of cathepsin K. Cathepsin K siRNA decreased Raptor protein expression and phosphorylation of OTUB1 and Src in Caki-1 (renal carcinoma), A549 (lung carcinoma), and DU145 (prostate carcinoma) cells, significantly (Fig. 1B). However, cathepsin K siRNA did not change Raptor mRNA expression (Fig. 1C). These data indicate that cathepsin K inhibition induces Raptor downregulation through dephosphorylation of OTUB1 at Y26.

### SHP2 is a key determinant of ODN-induced Raptor degradation through OTUB1 dephosphorylation

**Fig. 2:**
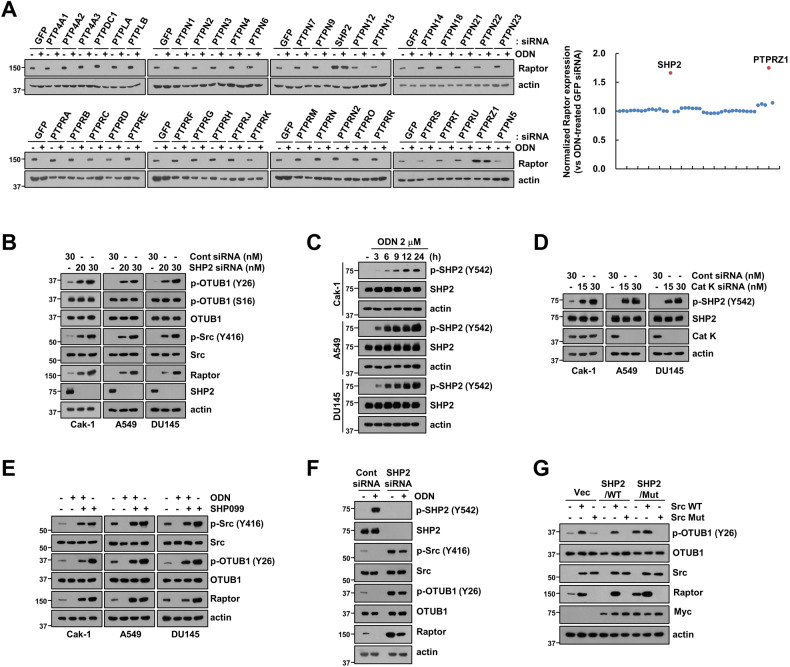
SHP2 phosphatase inhibits Src/OTUB1/Raptor axis by ODN. **A** Caki-1 cells were transfected with Cont siRNA or 41 tyrosine phosphatases siRNA followed by treatment with 2 μM ODN for 24 h. **B** Cancer cells (Caki-1, A549, and DU145) were transfected with Cont siRNA or SHP2 siRNA for 24 h. **C** Cancer cells (Caki-1, A549, and DU145) were treated with 2 μM ODN for the indicated periods. **D** Cancer cells (Caki-1, A549, and DU145) were transfected with Cont siRNA or Cat K siRNA for 24 h. **E** Cancer cells were pretreated with 10 μM SHP099 for 30 min, followed by treatment with 2 μM ODN for 24 h. **F** Caki-1 cells were transfected with Cont siRNA or SHP2 siRNA and followed by treatment with 2 μM ODN for 24 h. **G** Caki-1 were transiently co-transfected with a vector, SHP2 wild-type (WT), or SHP2 mutant (C459S) with Src WT or Src mutant (K295R/Y527F). Protein expression was determined using western blotting. The band intensity of Raptor protein was quantified by using ImageJ (**A**).

Previous reports suggested that Src kinase plays a major role in OTUB1 phosphorylation at Y26 and Raptor stabilization [15]. To identify the tyrosine phosphatase involved in OTUB1-dependent Raptor downregulation in ODN treatment, specific siRNAs for 41 tyrosine phosphatases were used. Among them, the knockdown of Src homology-2-containing protein tyrosine phosphatase 2 (SHP2) and protein tyrosine phosphatase receptor type (PTPRZ1) significantly inhibited ODN-induced Raptor downregulation (Fig. 2A). As PTPRTZ1 is mainly overexpressed in the central nervous system and glioma [19, 20], we chose SHP2 for further investigation. In various cancer cell lines, SHP2 was silenced using two different concentrations of siRNA. Interestingly, SHP2 depletion increased Raptor expression and OTUB1 (Y26) and Src (Y416) phosphorylation in a dose-dependent manner (Fig. 2B). However, phosphorylation of OTUB1 at serine 16 (S16) was unchanged in SHP2 siRNA-treated cells (Fig. 2B). These data suggest that SHP2 tyrosine phosphatase contributes to Raptor destabilization via OTUB1-Y26 dephosphorylation.

We further investigated the mechanisms involved in ODN-induced inhibition of OTUB1 phosphorylation at Y26. As SHP2 is a crucial tyrosine phosphatase in ODN-induced Raptor downregulation, we examined the role of SHP2 in the pathway. Phosphorylation of SHP2 at Y542 is an indicator of its activity [21], and ODN and siRNA-mediated cathepsin K knockdown phosphorylated SHP2 at Y542 residue (Fig. 2C, D). Figure 2E, F demonstrated that both SHP2 inhibitor (SHP099) and SHP2 knockdown alone induced the increase of Raptor and phosphorylation of Src (Y416) and OTUB1 (Y26), whereas ODN treatment induced Raptor downregulation and dephosphorylation of Src (Y416) and OTUB1 (Y26). To investigate the molecular mechanisms underlying OTUB1 phosphorylation, we used the SHP2 catalytic-dead mutant (C459S) and Src catalytic-dead mutant (K295R and Y527F). Mutation of SHP2 increased OTUB1 phosphorylation at Y26 thereby inducing Raptor protein expression. Moreover, the Src mutant inhibited SHP2 mutant-induced OTUB1 phosphorylation and Raptor upregulation (Fig. 2G), indicating that Src activation is required for SHP2 mutant-mediated OTUB1 phosphorylation and Raptor upregulation. Therefore, cathepsin K inhibition-mediated Raptor downregulation was mediated by SHP2 activation.

### Deletion of SHP2 inhibits ODN-induced mitochondrial dysfunction

**Fig. 3:**
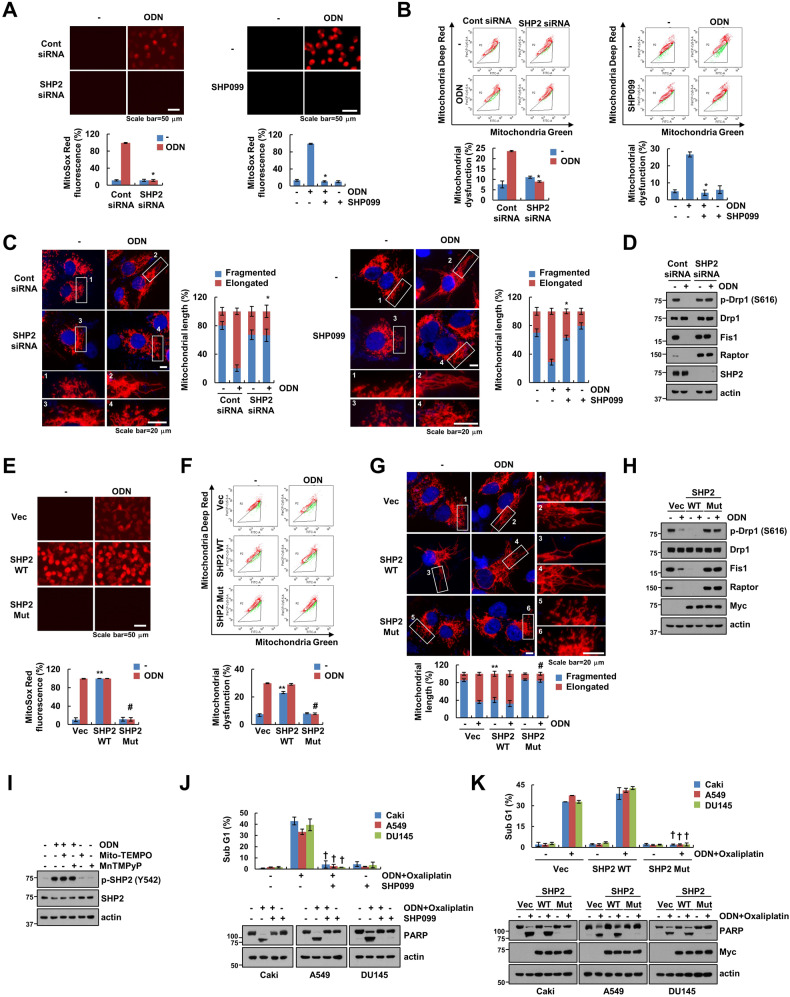
Involvement of phosphorylated SHP2 in ODN-mediated mitochondrial fusion and ROS. **A**–**D** Caki-1 cells were transfected with Cont siRNA or SHP2 siRNA followed by treatment with 2 μM ODN for 24 h (left panel). Caki-1 cells were pretreated with 10 μM SHP099 for 30 min and then treated with 2 μM ODN for 24 h (right panel). **E**–**H** Caki-1 cells were transfected with vector, SHP2 WT or SHP2 mutant (C459S) and treated with 2 μM ODN for 24 h. **I** Caki-1 cells were pretreated with 2 μM Mito-TEMPO or 10 μM MnTMPyP for 30 min, followed by treatment with 2 μM ODN for 24 h. **J** Caki-1 cells were pretreated with 10 μM SHP099 for 30 min, followed by incubation with 2 μM ODN and 25 μM oxaliplatin for 24 h. **K** Caki-1 cells were transfected with vector, SHP2 WT or SHP2 mutant (C459S) and followed by incubation with 2 μM ODN and 25 μM oxaliplatin for 24 h. Mitochondrial ROS production was assessed after MitoSOX Red staining using microscopy and flow cytometry (**A**, **E**). Flow cytometry was used to detect the fluorescence intensity after mitochondrial damage (**B**, **F**). Representative confocal images of Caki-1/pDsRed2-mito cells. The nuclei were stained with DAPI, and the length of the mitochondria was measured using ZEN3.4 (**C**, **G**). Apoptosis and protein expression were measured using flow cytometry (**J**, **K**) and western blotting (**D**, **H**–**K**). The values in the graphs represent the mean ± SD of three independent experiments. ^*^P < 0.01 compared to ODN. ^**^P < 0.01 compared to the vector-transfected cells. ^#^*P* < 0.01 compared to ODN-treated vector-transfected cells. ^†^*P* < 0.05 compared to combinations of ODN and oxaliplatin.

Our previous studies suggested that ODN induces mitochondrial ROS production and mitochondrial fusion [12]. Therefore, we explored the role of SHP2 in ODN-induced mitochondrial dysfunction. Depletion of SHP2 (by using pharmacological inhibitor SHP099 and siRNA) markedly prevented ODN-induced mitochondrial ROS production, mitochondrial dysfunction, and mitochondrial fusion (Fig. 3A–C). ODN decreased phosphorylation of Drp1 at S616 and expression of Fis1, the key regulators of mitochondrial fission, but these effects were blocked by SHP2 knockdown (Fig. 3D). Moreover, similar results were obtained in ODN-treated SHP2 mutant-transfected cells (Fig. 3E–H). In contrast to the SHP2 mutant, overexpression of SHP2 wild-type (WT) induced mitochondrial ROS, mitochondrial dysfunction, mitochondrial fusion, dephosphorylation of Drp1 S616, and downregulation of Fis1 (Fig. 3E–H). Next, we investigated the relationship between mitochondrial ROS production and SHP2 activation by using specific mitochondrial ROS inhibitors (Mito-TEMPO and MnTMPyp). Results suggested that mitochondrial ROS inhibitors did not inhibit ODN-induced SHP2 phosphorylation (Fig. 3I). Since we previously reported that ODN-mediated Raptor downregulation enhances sensitivity to anti-cancer drugs [12], we examined the involvement of SHP2 activation in apoptosis induced by combination treatment (ODN plus oxaliplatin). The SHP2 inhibitor and SHP2 mutant prevented ODN plus oxaliplatin-mediated apoptosis in all tested cancer cell lines (Fig. 3J, K). Therefore, it can be suggested that SHP2 activation is upstream in the signaling pathway involved in ODN-induced Raptor downregulation and mitochondrial dysfunction, eventually enhancing sensitivity to anti-cancer drugs.

### Overexpression of Raptor alleviates ODN-induced mitochondrial dysfunction

**Fig. 4:**
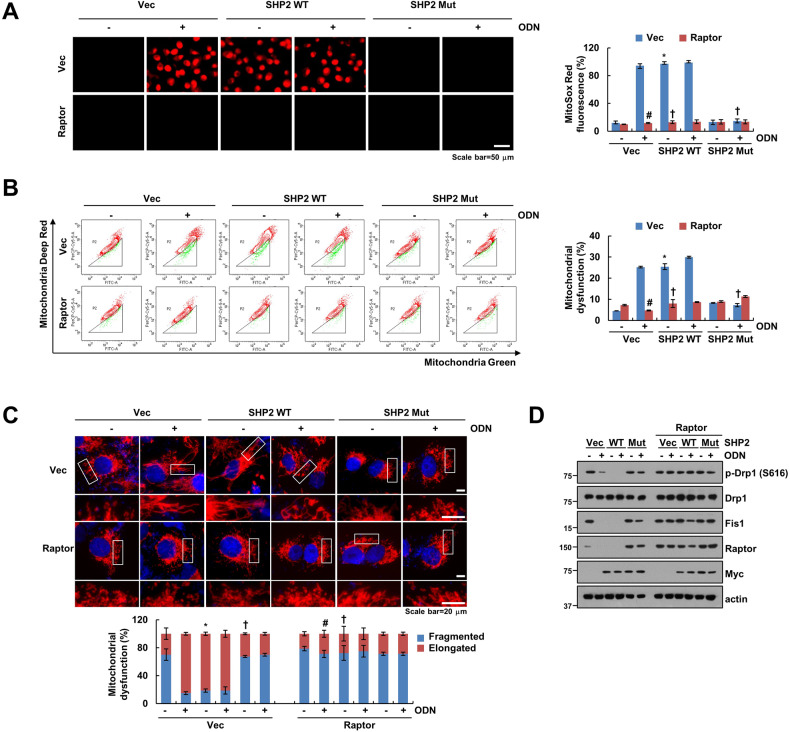
Upregulation of Raptor alleviates ODN-induced mitochondrial dysfunction. **A**–**D** Caki-1 were transiently transfected with an SHP2 WT or SHP2 mutant (C459S) with/without Raptor. Mitochondrial ROS production was assessed after MitoSOX Red staining using microscopy and flow cytometry (**A**). Flow cytometry was used to detect the fluorescence intensity after mitochondrial damage (**B**). Representative confocal images of Caki-1/pDsRed2-mito cells. The nuclei were stained with DAPI, and the length of the mitochondria was measured using ZEN3.4 (**C**). Protein expression was determined using western blotting (**D**). The values in the graphs represent the mean ± SD of three independent experiments. ^#^*P* < 0.01 compared to the ODN in vector-treated cells. ^*^P < 0.01 compared to the vec-transfected cells. ^†^*P* < 0.01 compared to the ODN in SHP2 WT-transfected cells.

In this study, we also investigated the role of Raptor in ODN-induced mitochondrial dysfunction via SHP2 activation. Overexpression of Raptor significantly interrupted the ODN- and SHP2 overexpression-induced mitochondrial ROS production and mitochondrial dysfunction (Fig. 4A, B). In addition, Raptor overexpression decreased mitochondrial fusion in ODN-treated and SHP2-overexpressed cells (Fig. 4C). Furthermore, ODN- and SHP2 overexpression-induced dephosphorylation of Drp1 at S616 and downregulation of Fis1 were blocked by Raptor overexpression (Fig. 4D). These data suggest that downregulation of Raptor plays a critical role in ODN-induced mitochondrial dysfunction via the SHP2/OTUB1 axis signaling pathway.

### Spleen tyrosine kinase (Syk) and Zeta-chain-associated protein (ZAP-70) tyrosine kinases play role in ODN-mediated SHP2/OTUB1 axis signaling pathway

**Fig. 5:**
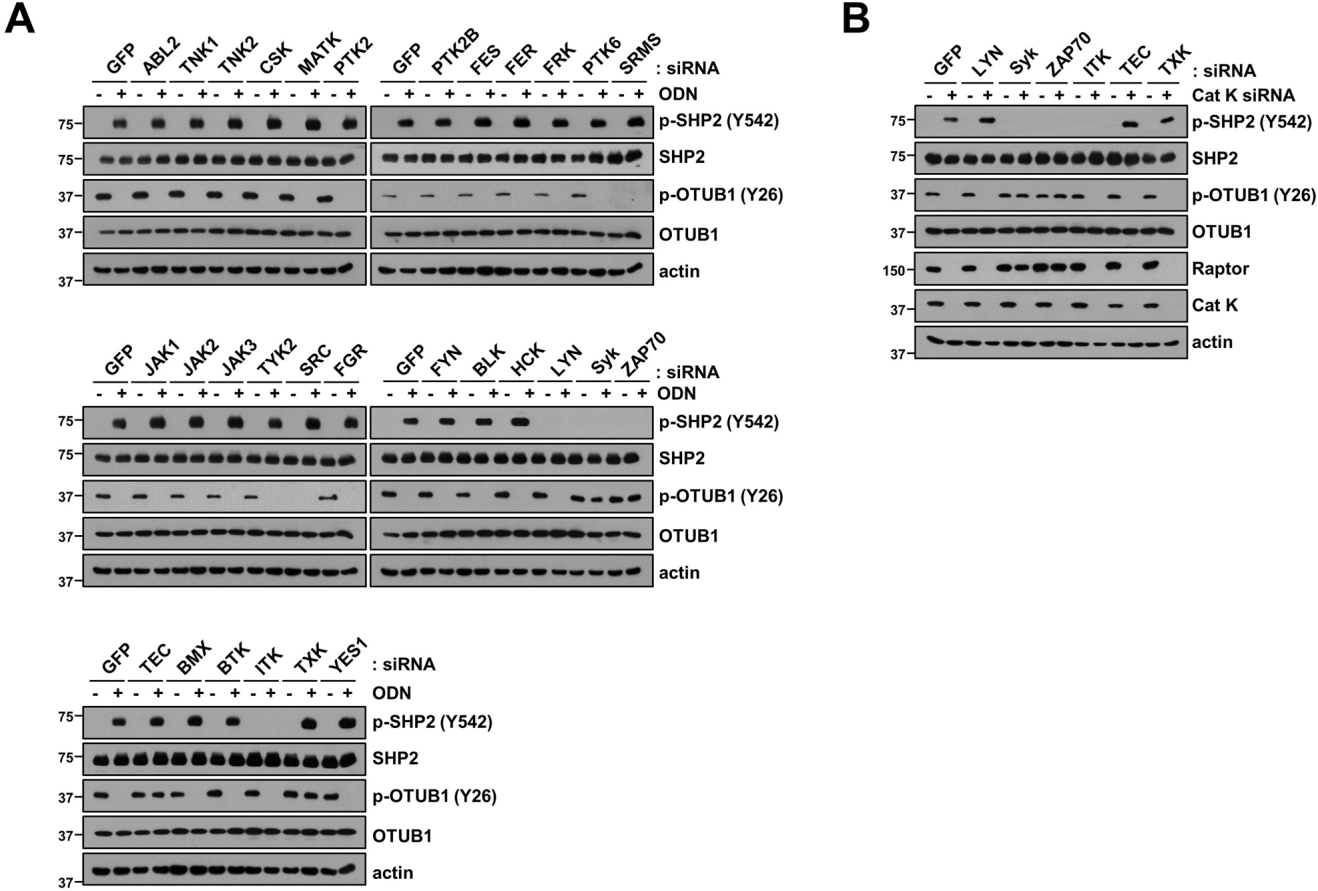
Identification of non-receptor tyrosine kinase for SHP2 phosphorylation during cathepsin K inhibition. **A** Caki-1 cells were transfected with Cont siRNA or 30 non-receptor tyrosine kinases siRNA and treated with 2 μM ODN for 24 h. **B** Caki-1 were transiently co-transfected with a Cont siRNA or 6 tyrosine kinases siRNA with/without Cat K siRNA. Protein expression was determined using western blotting.

To identify specific non-receptor tyrosine kinases that can activate SHP2, we co-transfected 30 different siRNAs that could knock down the respective non-receptor tyrosine kinases in the presence or absence of ODN treatment. Knockdown of four non-receptor tyrosine kinases (Lyk, Syk, ZAP70, and ITK) inhibited SHP2-Y542 phosphorylation (Fig. 5A). Since we focused on SHP2-mediated phosphorylation of OTUB1 at Y26, we excluded LYN and ITK, as siRNA of LYN and ITK still dephosphorylated OTUB1 at Y26 in ODN-treated cells (Fig. 5A). We further confirmed that Syk and ZAP70 likely affect SHP2-mediated OTUB1-Y26 phosphorylation in cathepsin K siRNA-treated cells. As shown in Fig. 5B, both Syk and ZAP70 knockdown decreased the phosphorylation of SHP2 (Y542) and increased the phosphorylation of OTUB1 (Y26) in cathepsin K siRNA-treated cells. Therefore, these data suggest that cathepsin K inhibition-mediated phosphorylation of SHP2 at Y542 (SHP2 activation) is regulated by Syk and ZAP70 non-receptor tyrosine kinases.

### Syk activation is a key determinant of Raptor degradation-mediated mitochondrial dysfunction by ODN

**Fig. 6:**
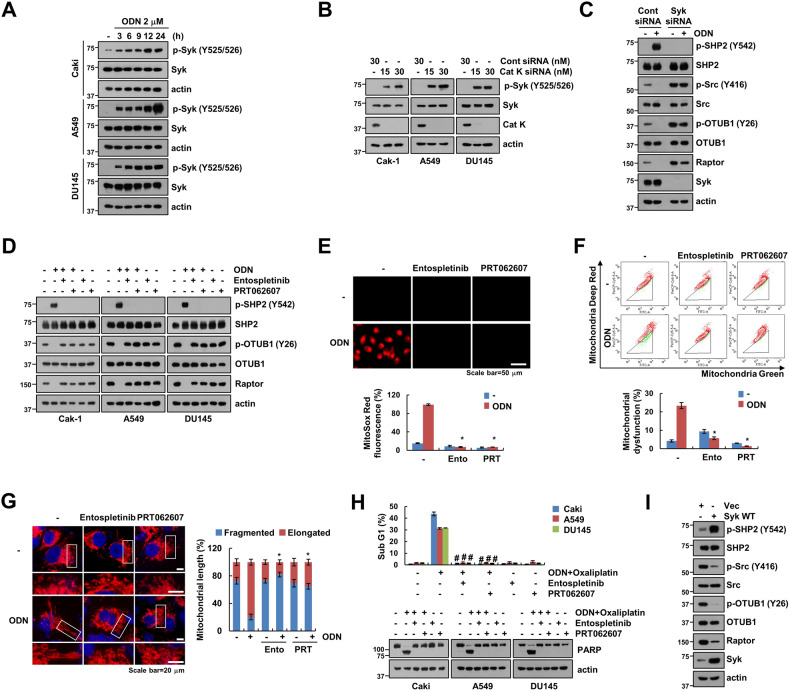
ODN-mediated Syk phosphorylation induces mitochondria dysfunction via SHP1/OTUB1/Raptor axis. **A** Cancer cells (Caki-1, A549, and DU145) were treated with 2 μM ODN for the indicated periods. **B** Cancer cells (Caki-1, A549, and DU145) were transfected with Cont siRNA or Cat K siRNA for 24 h. **C** Caki-1 cells were transfected with Cont siRNA or Syk siRNA followed by treatment with 2 μM ODN for 24 h. **D**–**G** Caki-1 cells were pretreated with 10 μM Entospletinib or 2 μM PRT062607 for 30 min, followed by treatment with 2 μM ODN for 24 h. **H** Caki-1 cells were pretreated with 10 μM Entospletinib or 2 μM PRT062607 for 30 min, followed by incubation with 2 μM ODN and 25 μM oxaliplatin for 24 h. **I** Caki-1 cells were transfected with vector and Syk WT for 24 h. Mitochondrial ROS production was assessed after MitoSOX Red staining using microscopy and flow cytometry (**E**). Flow cytometry was used to detect the fluorescence intensity after mitochondrial damage (**F**). Representative confocal images of Caki-1/pDsRed2-mito cells. The nuclei were stained with DAPI, and the length of the mitochondria was measured using ZEN3.4 (**G**). Apoptosis and protein expression were measured using flow cytometry (**H**) and western blotting (**A**–**D**, **H**, **I**). The values in the graphs represent the mean ± SD of three independent experiments. ^*^P < 0.01 compared to the ODN. ^#^*P* < 0.01 compared to combinations of ODN and oxaliplatin.

Syk and ZAP-70 are structurally and functionally homologous tyrosine kinases that play a role in hematopoietic and non-hematopoietic cells [22, 23]. Moreover, Coopman et al. reported that Syk is a potent modulator of epithelial cell growth and a potential tumor suppressor in human breast carcinomas. [24]. The intrinsic enzymatic activity of Syk is superior to that of ZAP-70 and such a distinction relates to structural variations in the catalytic domain [25]. Therefore, we focused on the functional role of Syk in ODN-induced Raptor downregulation. Previous results indicate that phosphorylation of Syk at Y525 and Y526 tyrosine residues is essential for its function [26]. Our data showed that ODN and cathepsin K siRNA significantly increases the phosphorylation of Syk at the Y525/Y526 residues (Fig. 6A, B). We observed that siRNA and pharmacological inhibitors (Entospletinib and PRT062607) of Syk reduced ODN-induced SHP2 phosphorylation (Y542), OTUB1 dephosphorylation (Y26), and Raptor downregulation (Fig. 6C, D). In addition, Syk inhibitors markedly prevented ODN-induced mitochondrial ROS production, mitochondrial dysfunction, and mitochondrial fusion (Fig. 6E–G). Pharmacologic inhibitors of Syk also prevented ODN plus oxaliplatin-induced apoptosis and PARP cleavage (Fig. 6H). Furthermore, overexpression of Syk induced phosphorylation of SHP2 (Y542), dephosphorylation of Src (Y416) and OTUB1(Y26), and downregulation of Raptor (Fig. 6I). These results indicate that Syk activation plays a critical role in cathepsin K inhibition-mediated Raptor downregulation and mitochondrial dysfunction.

### Syk expression positively correlates with SHP2 expression in human RCC patient tissues

**Fig. 7:**
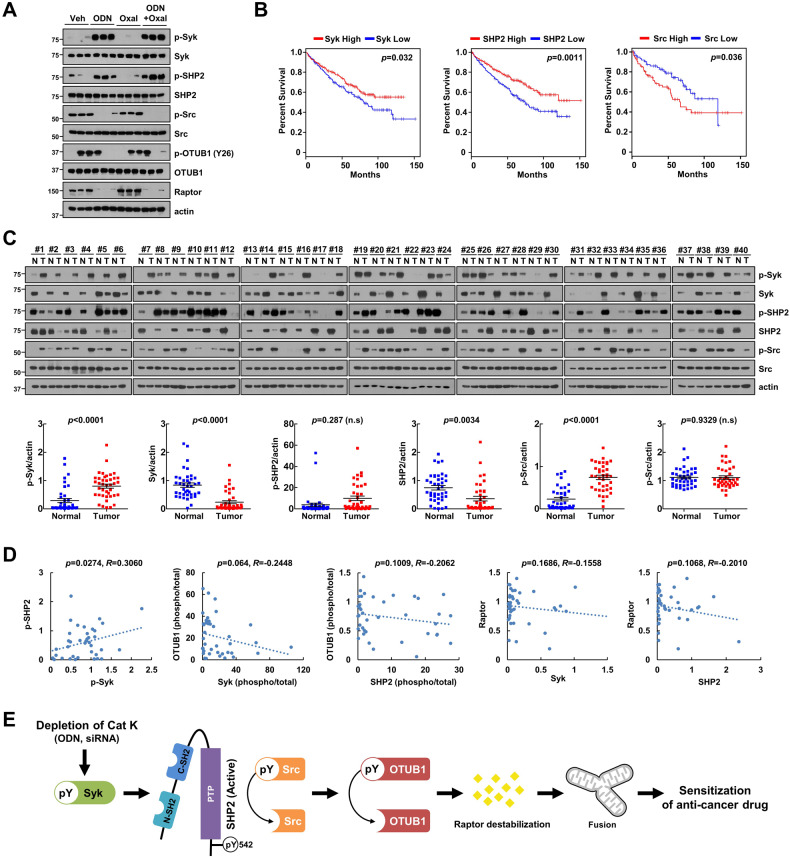
Correlation analysis of Syk/SHP2/OTUB1/Raptor in RCC patients. **A** Mice were treated with 5 mg/kg ODN, 5 mg/kg oxaliplatin, a combination, or vehicle for 20 days. Protein expression was determined using western blotting. **B** Survival analysis of the TCGA patient cohort in Oncolnc. Patients from the TCGA cohort were divided into high and low gene-expressing groups based on the average expression. **C** Examination of protein expression in 40 unpaired primary renal tumor tissues and the corresponding normal adjacent tissues. **D** Correlation analysis of phospho-Syk/phospho-SHP2, Syk/OTUB1, SHP2/OTUB1, Syk/Raptor, or SHP2/Raptor. **E** Scheme of the mechanisms of mitochondrial dysfunction by ODN-mediated Src/OTUB1/Raptor axis through phosphorylation of Syk/SHP2.

To present the possibility of Syk and SHP2 as therapeutic targets in malignant development, we examined the colony formation assay and XTT assay. Combined treatment with ODN plus oxaliplatin decreased colony formation and proliferation, which was impeded by the inactivation of SHP2 (SHP099 and mutated overexpression) or Syk (Entospletinib and PRT062607) (Supplementary Fig. S1A–C). Interestingly, SHP2 overexpression alone inhibited colony formation and proliferation, although the sub-G1 population did not increase (Supplementary Fig. S1B and Fig. 3K). Moreover, ODN plus oxaliplatin treatment diminished cancer cell proliferation in SHP2 WT-transfected cells (Supplementary Fig. S1B). Therefore, these results demonstrate the involvement of Syk and SHP2 in tumor cell growth. Previously, we reported the synergistic effect of ODN on anti-cancer drugs in an in vivo xenograft model [12, 13]. To prove the relevance of ODN-mediated Syk/SHP2/Src/OTUB1 regulating mechanisms in vivo experiments, we detected protein expression using previously obtained lysates from in vivo xenograft models [13]. ODN alone and combined treatment with ODN plus oxaliplatin induced phosphorylation of Syk and SHP2, dephosphorylation of Src and OTUB1, and downregulation of Raptor (Fig. 7A).

Following Kaplan–Meier plotter analysis, we analyzed the overall survival of patients with human renal clear carcinoma (RCC) and prognostic relationships among Syk, SHP2, and Src. Increased expression of Syk and SHP2 was associated with favorable prognosis, whereas increased expression of Src was associated with poor prognosis in RCC patients (Fig. 7B). Next, we analyzed phosphorylation and total expression of Syk, SHP2, and Src proteins in 40 specimens of RCC tissues, and quantified that 80% of Syk (32/40) and 72.5% of SHP2 (29/40) were lower, whereas 85% of phospho-Src (34/40) was higher compared to adjacent normal tissue (Fig. 7C). Recently, we reported that phospho-OTUB1 and Raptor are overexpressed in RCC tumor tissues, and identified the positive correlation of between two proteins [15]. Therefore, we investigated the correlation among the Syk/SHP2/OTUB1/Raptor on the basis of previous data on tumor tissues with RCC. These results indicated a positive correlation between phospho-Syk and phospho-SHP2, whereas OTUB1 and Raptor showed an inverse correlation with Syk and SHP2 (Fig. 7D).

Our findings demonstrate that cathepsin K inhibition induces OTUB1 dephosphorylation-mediated Raptor destabilization through Syk-SHP2 activation leading to an increase in mitochondrial dysfunction and anti-cancer sensitivity.

## Discussion

In this study, we demonstrated that a cathepsin K inhibitor (ODN) induces Raptor destabilization and enhances mitochondrial dysfunction in cancer cells. ODN-induced Raptor degradation was regulated by OTUB1-Y26 dephosphorylation, which was caused by SHP2 activation mediated by Syk non-receptor tyrosine kinases. ODN induced phosphorylation of SHP2 (Y542) and dephosphorylation of Src (Y416) and OTUB1 (Y26) resulting in Raptor destabilization. Interestingly, Syk-mediated SHP2 activation increased mitochondrial ROS production and dysfunction in ODN-treated cells. Together, the Syk/SHP2/Src/OTUB1 axis contributed to ODN-induced anti-cancer drug sensitization via Raptor degradation (Fig. 7E).

Our previous study suggested that cathepsin K inhibition enhances anti-cancer drug-induced apoptotic cell death through the upregulation of USP27x-mediated Bim pro-apoptotic protein [12]. This study emphasizes Raptor downregulation as an upstream regulator of the anti-cancer drug enhancement effect, which is regulated by Src-dependent dephosphorylation of OTUB1 at Y26 [15]. In our previous studies, we found that ODN causes Raptor downregulation and OTUB1 induces Raptor degradation, hence, in this study, we investigated the detailed molecular mechanisms to between ODN and OTUB1/Raptor. Pharmacological and genetic inhibition of cathepsin K significantly decreased Raptor expression, resulting in dephosphorylation of Src and OTUB1 at the tyrosine residue (Fig. 1). Next, we examined the role of tyrosine phosphatase in regulating OTUB1 phosphorylation and discovered that SHP2 knockdown prevented ODN-mediated Raptor downregulation (Fig. 2A). SHP2, an oncogenic tyrosine phosphatase, is involved in multiple signaling pathways, including the Ras/Raf/MEK/ERK, PI3K/AKT, JAK/STAT, and PD-1/PD-L1 pathways [27]. In response to growth factors such as PDGF and FGF, phosphorylation of two tyrosine residues (Y542 and Y580) at the C-terminal of SHP2 provides binding sites for the Grb2/SOS [28]. Unbiased phosphoproteomic and biochemical analyses demonstrated that SHP2 activates several SRC-family kinases [29]. Interestingly, depletion of SHP2 phosphorylated OTUB1 (Y26) and Src (Y416), but not OTUB1 (S16) (Fig. 2B). Moreover, inhibition of cathepsin K (by ODN and siRNA) significantly increased SHP2 phosphorylation at Y542, and SHP2 inhibition (by using pharmacological inhibitor and siRNA) interrupted the ODN-mediated dephosphorylation of OTUB1 and Src, eventually stabilizing Raptor (Fig. 2C–F). In addition, the inactivation of SHP2 decreased ODN-induced mitochondrial ROS production, mitochondrial dysfunction, and mitochondrial fusion through Raptor stabilization (Figs. 3 and 4). Therefore, we proposed that activation of the SHP2 signaling pathway is responsible for cathepsin K inhibition-mediated Raptor destabilization.

We provided further convincing evidence to show that Syk modulates SHP2-Y542 phosphorylation and regulates ODN-mediated Raptor expression and mitochondrial fusion (Fig. 6C–G). Previous studies showed that Syk is an attractive target for the treatment of immune disorders and solid tumors [30, 31]. Proximity biotinylation experiments have identified Syk as a kinase implicated in the phosphorylation of Deptor, an endogenous inhibitor of mTOR, in an ephrin receptor-dependent manner [32]. Recently, we reported that ODN induces DNA damage via Raptor downregulation-mediated mitochondrial ROS generation [14]. Previous reports suggested that checkpoint kinase 1 (CHK1) is activated in response to genotoxic damage [33], and phosphorylation of Syk on S295 by CHK1 promotes tumorigenesis in hepatocellular carcinoma via Syk degradation, but these signaling pathways were inconsistent with our results [34]. The Syk/SHP2/OTUB1/Raptor signaling axis acted upstream of mitochondrial ROS generation and mitochondrial damage in our study (Fig. 6E, F). ODN phosphorylated Syk at tyrosine residues (Y525/Y526) that did not impact Syk protein expression. This led to the SHP2-dependent dephosphorylation of Src and OTUB1 (Fig. 6A, D), and ODN-induced Syk activation increased sensitivity to the anti-cancer drug through regulation of SHP2/OTUB1/Raptor axis (Fig. 6H). Our findings suggested that Syk activation acts as an upstream signaling trigger for cathepsin K inhibition-mediated Raptor downregulation and mitochondrial dysfunction. Further studies are required to elucidate how cathepsin K inhibition causes Syk activation.

In conclusion, this study demonstrated that cathepsin K inhibition-mediated Raptor downregulation is regulated by Syk/SHP2/Src/OTUB1 signaling axis. The pharmacological and genetic inhibition of cathepsin K enhanced the sensitivity to anti-cancer drugs. Therefore, cathepsin K inhibitor may be an effective candidate for anti-cancer adjuvants.

## Supplementary information

Supplementary information

Original Data File

aj-checklist

Supplementary Figure S1

## Data Availability

All data are available from the corresponding author upon reasonable request.

## Materials availability

All materials are available from the corresponding author upon reasonable request.
