# Supplementary material for: Cathepsin K inhibition induces Raptor destabilization and mitochondrial dysfunction via Syk/SHP2/Src/OTUB1 axis-mediated signaling
Source: Cell Death Dis. 2023 Jun 17;14(6):366. doi: 10.1038/s41419-023-05884-z (PMC10276854; doi:10.1038/s41419-023-05884-z)
Supplement: Supplementary file 1 — Supplementary information [file 41419_2023_5884_MOESM1_ESM.docx]

**Supplementary Information**

**Supplementary Figure legends**

**Supplementary Tables S1-2**

**Supplementary Methods**

**Fig. S1 Inactivation of Syk and SHP2 prevents ODN plus oxaliplatin-mediated decrease of cancer growth. A** Caki-1 cells were pretreated with 10 μM SHP099 for 30 min, followed by incubation with 2 μM ODN and 25 μM oxaliplatin. **B** Caki-1 cells were transfected with vector, SHP2 WT or SHP2 mutant (C459S) and followed by incubation with 2 μM ODN and 25 μM oxaliplatin. **C** Caki-1 cells were pretreated with 10 μM Entospletinib or 2 μM PRT062607 for 30 min, followed by incubation with 2 μM ODN and 25 μM oxaliplatin. Colony formation was measured using crystal violet staining (upper panel). Cell viability was determined using XTT assay (lower panel).

**Table S1. The information of chemicals or siRNA for silencing gene.**

| Chemicals | Source | Cat # |
| --- | --- | --- |
| Odanacatib (ODN) | Cayman chemical | Cat# 21466 |
| SHP099 HCl | Selleckchem | Cat# S8278 |
| Entospletinib | Selleckchem | Cat# S7523 |
| PRT062607 HCl | Selleckchem | Cat# S8032 |
| Mito-TEMPO | Sigma-Aldrich | Cat# SML0737 |
| MnTMPyP | Sigma-Aldrich | Cat# 475872 |
| Oxaliplatin | Sigma-Aldrich | Cat# O9512 |
| Name | Sequence (5’-3’) | |
| GFP (control) siRNA | GUUCAGCGUGUCCGGCGAG | |
| Cathepsin K (Cat K) siRNA | Santa Cruz Biotechnology (Cat# sc-29936) | |

**Table S2. The list of antibodies.**

| Antibodies | Source | Cat # |
| --- | --- | --- |
| β-actin | Sigma-Aldrich | Cat# A2228 |
| p-DRP1 (S616) | Cell Signaling Technology | Cat# 3455 |
| DRP1 | Cell Signaling Technology | Cat# 8570 |
| Fis1 | Santa Cruz Biotechnology | Cat# sc-376447 |
| Myc-Tag | Cell Signaling Technology | Cat# 2272 |
| OTUB1 | Santa Cruz Biotechnology | Cat# sc-130458 |
| PARP | Cell Signaling Technology | Cat# 9542 |
| Raptor | Cell Signaling Technology | Cat# 2280 |
| Src | Merck Millipore | Cat# 05-184 |
| p-Src (Y416) | Cell Signaling Technology | Cat# 2101 |
| Cathepsin K | Abcam | Cat# ab207086 |
| p-OTUB1 (S16) | Affinity Biosciences | Cat# AF3558 |
| p-SHP2 (Y542) | Cell Signaling Technology | Cat# 3751 |
| SHP2 | Cell Signaling Technology | Cat# 3752 |
| p-SyK (Y525/526) | Cell Signaling Technology | Cat# 2710 |
| SyK | Cell Signaling Technology | Cat# 80460 |

**Colony formation assay**

The cells (5 × 10^4^) were seeded in a 12-well culture plates, and then treated with each drug, or transiently transfected with each plasmid. After 5 days, the cells were fixed, stained with 0.5% crystal violet solution, and visualized by a digital camera.

**Cell viability analysis**

Cell viability was measured using the WelCountTM Cell Viability Assay Kit (Welgene, Daegu, Korea). Caki-1 cells were treated with each drug, or transiently transfected with each plasmid. After 24 h, XTT reagent and 1% PMS reagent were added to each well, and cells were incubated for 1 h at 37 °C, thereafter. Cell viability was measured with a multiwell plate reader using an excitation filter at 450 nm.
