# Supplementary material for: Cathepsin K inhibition induces Raptor destabilization and mitochondrial dysfunction via Syk/SHP2/Src/OTUB1 axis-mediated signaling
Source: Cell Death Dis. 2023 Jun 17;14(6):366. doi: 10.1038/s41419-023-05884-z (PMC10276854; doi:10.1038/s41419-023-05884-z)

Figure 1A

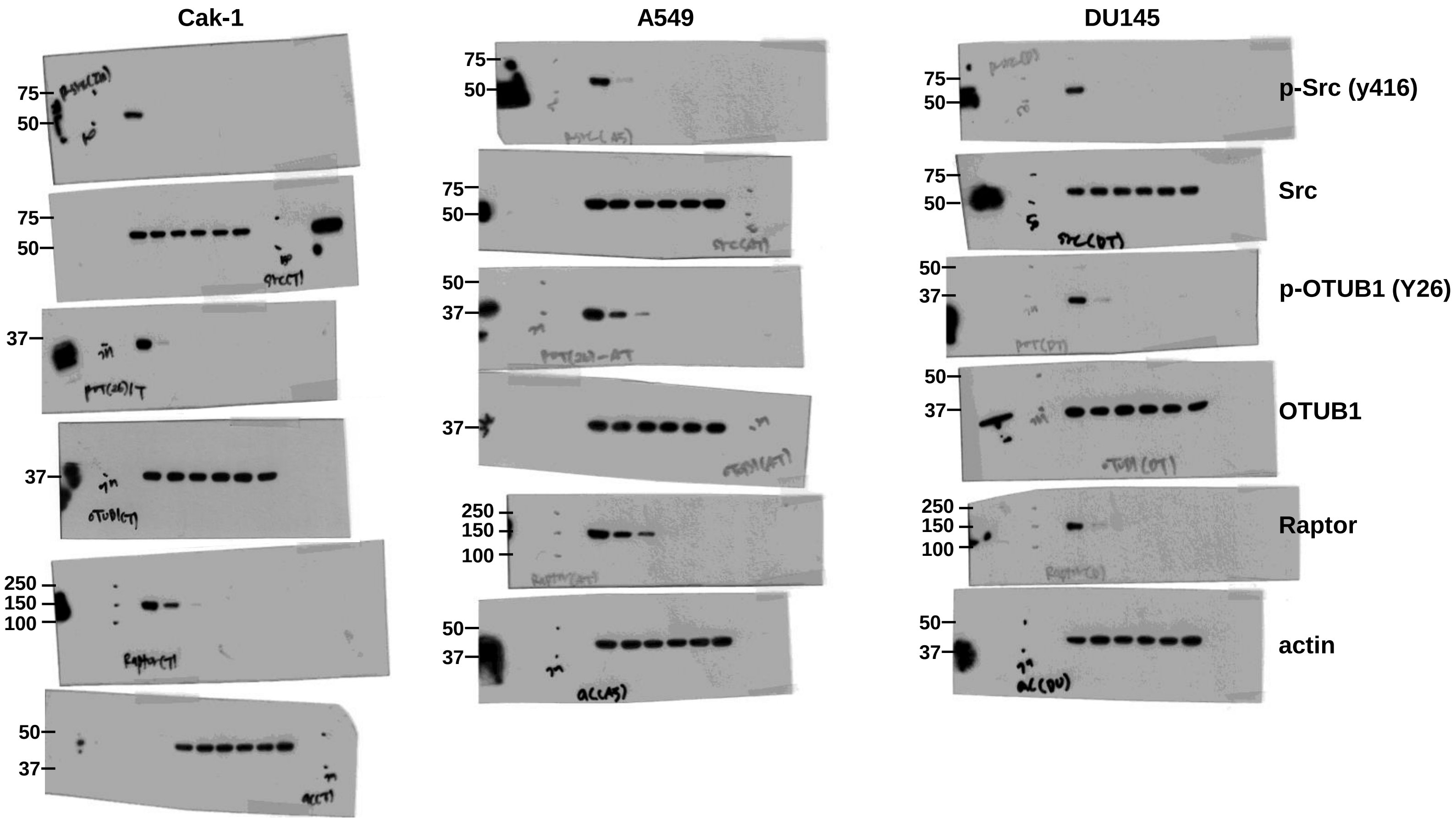

Figure 1B

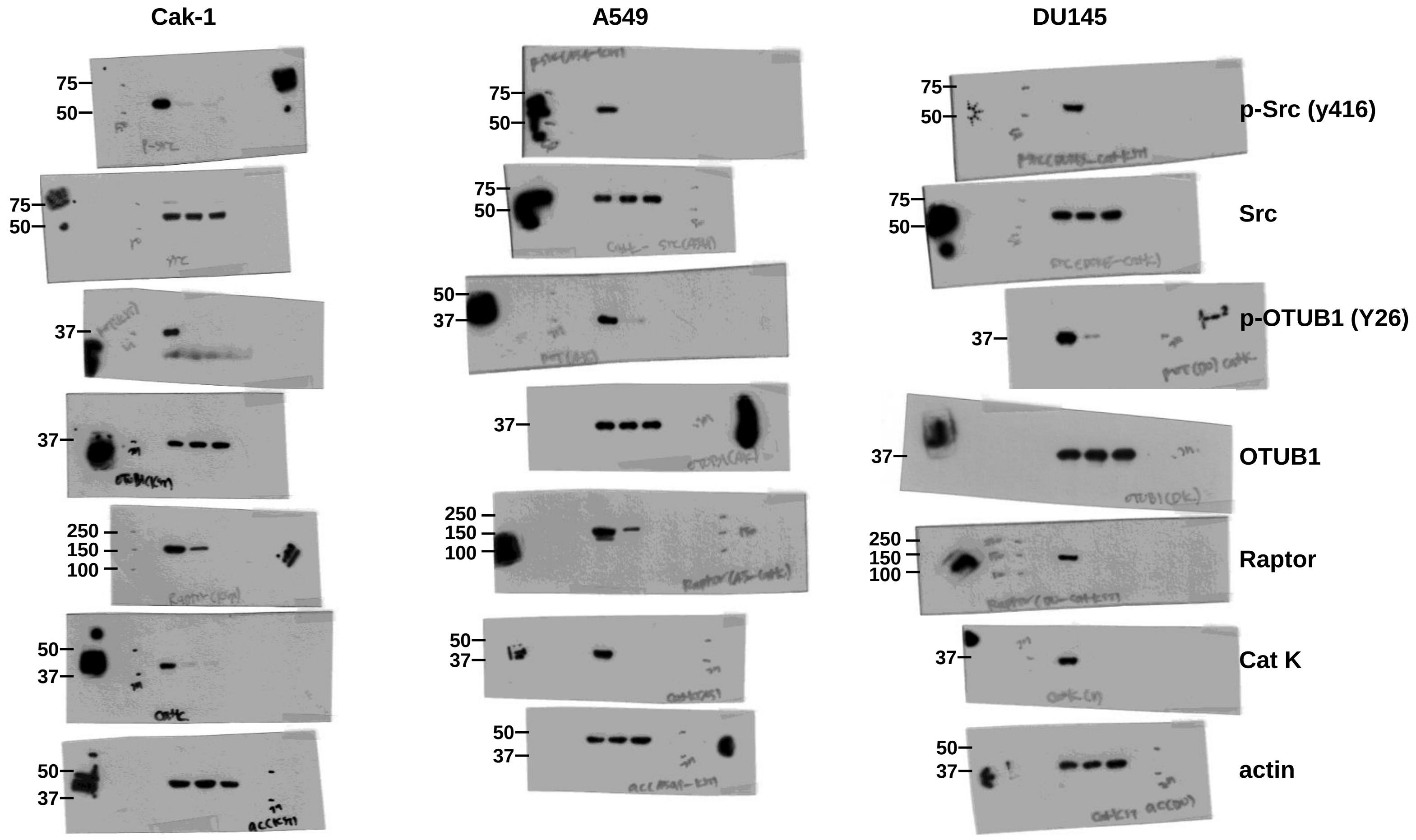

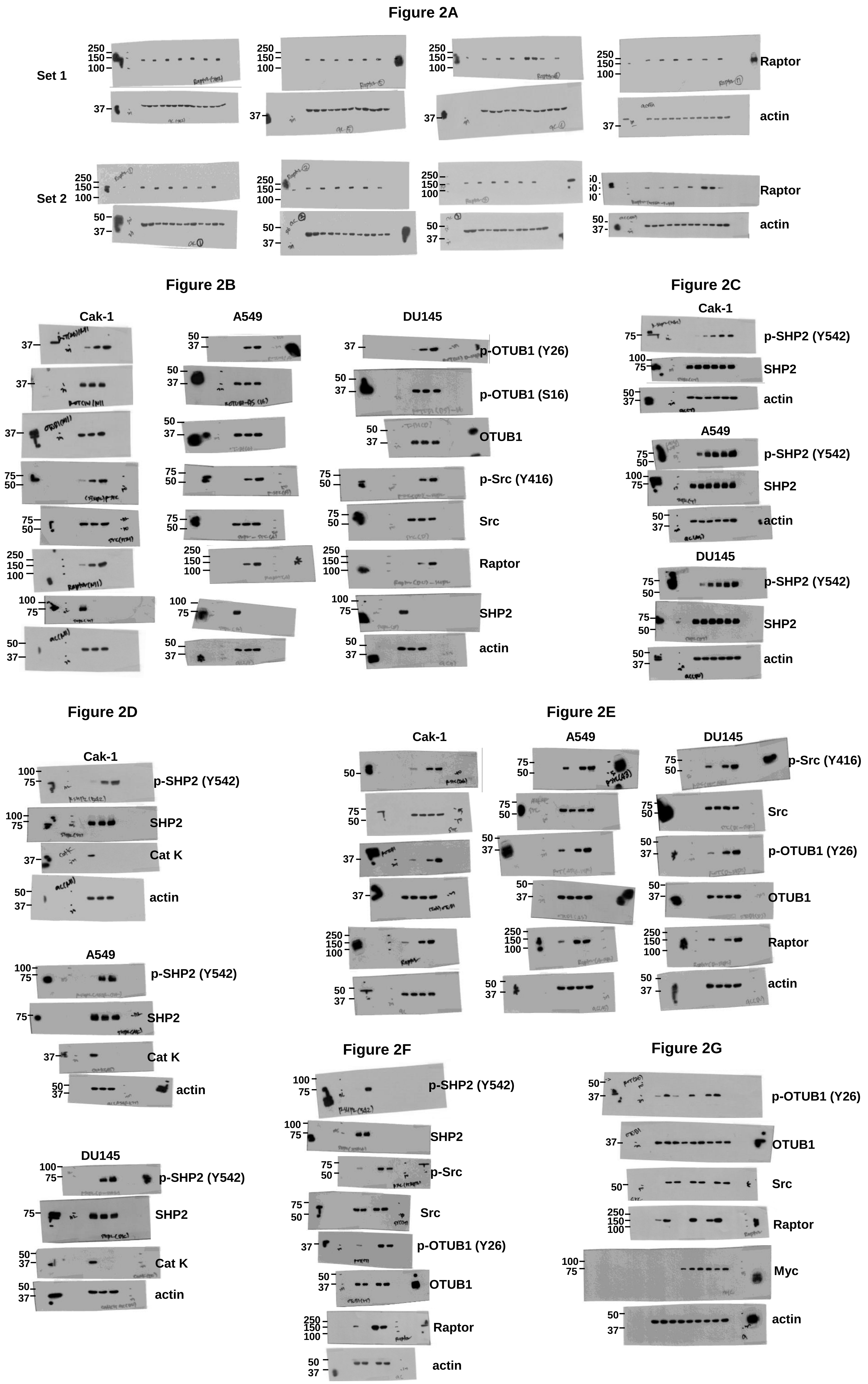

Figure 2A
Set 1
Set 2
250
150
100
50
37
Raptor
actin
Figure 2B
Cak-1
A549
DU145
p-OTUB1 (Y26)
p-OTUB1 (S16)
OTUB1
p-Src (Y416)
Src
Raptor
SHP2
actin
Figure 2C
Cak-1
A549
DU145
p-SHP2 (Y542)
SHP2
actin
Figure 2D
Cak-1
A549
DU145
p-SHP2 (Y542)
SHP2
Cat K
actin
Figure 2E
Cak-1
A549
DU145
p-Src (Y416)
Src
p-OTUB1 (Y26)
OTUB1
Raptor
actin
Figure 2F
p-SHP2 (Y542)
SHP2
p-Src
Src
p-OTUB1 (Y26)
OTUB1
Raptor
actin
Figure 2G
p-OTUB1 (Y26)
OTUB1
Src
Raptor
Myc
actin

Figure 3D

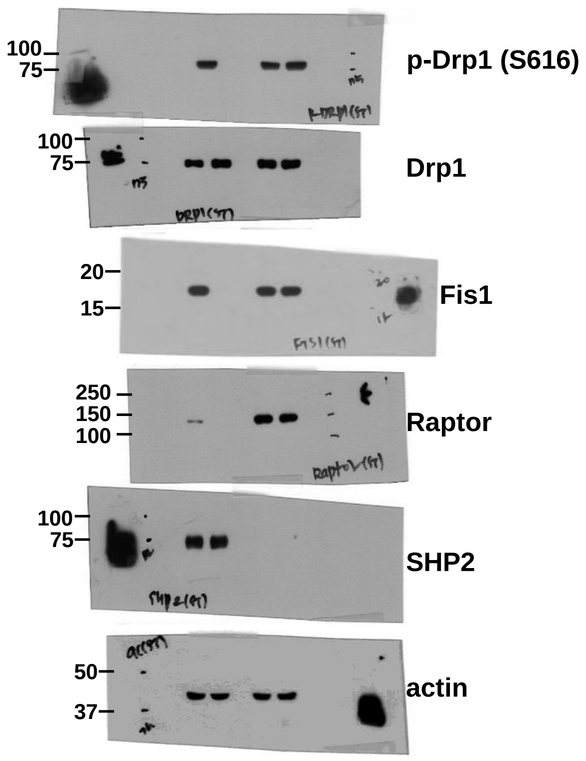

Figure 3H

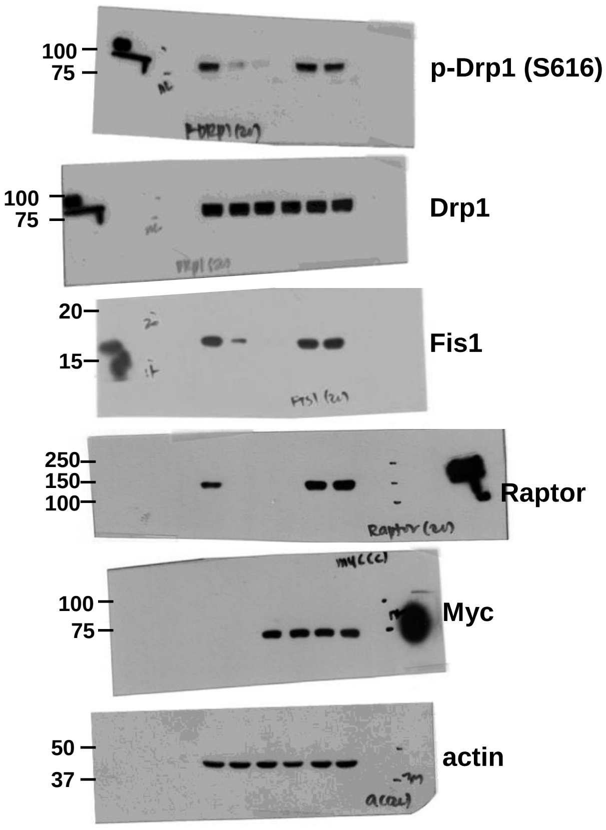

Figure 3I

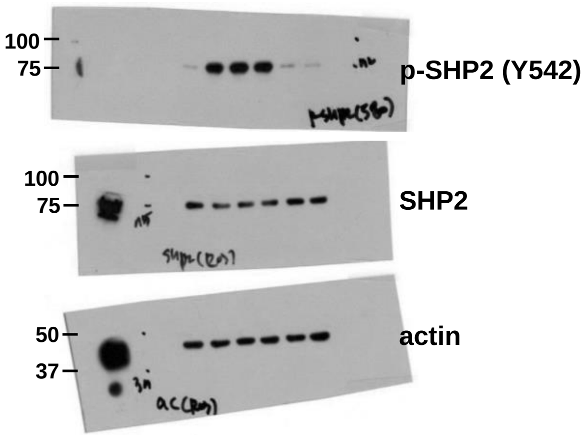

Figure 3J

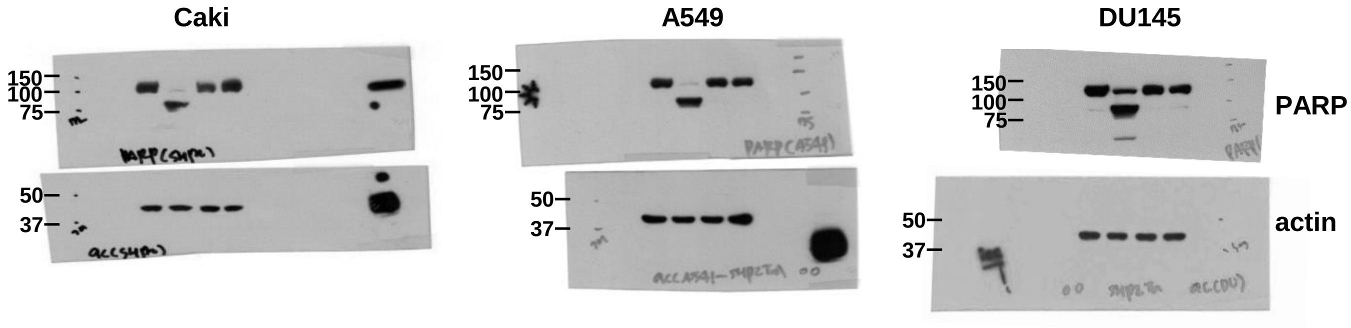

Figure 3K

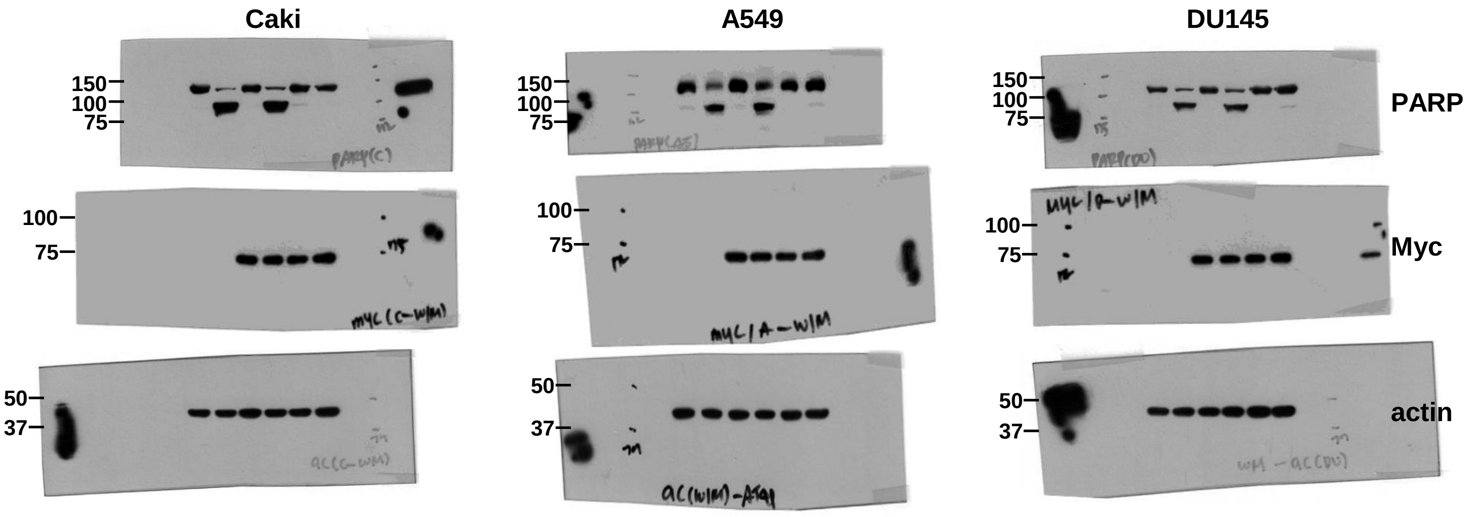

Figure 4D

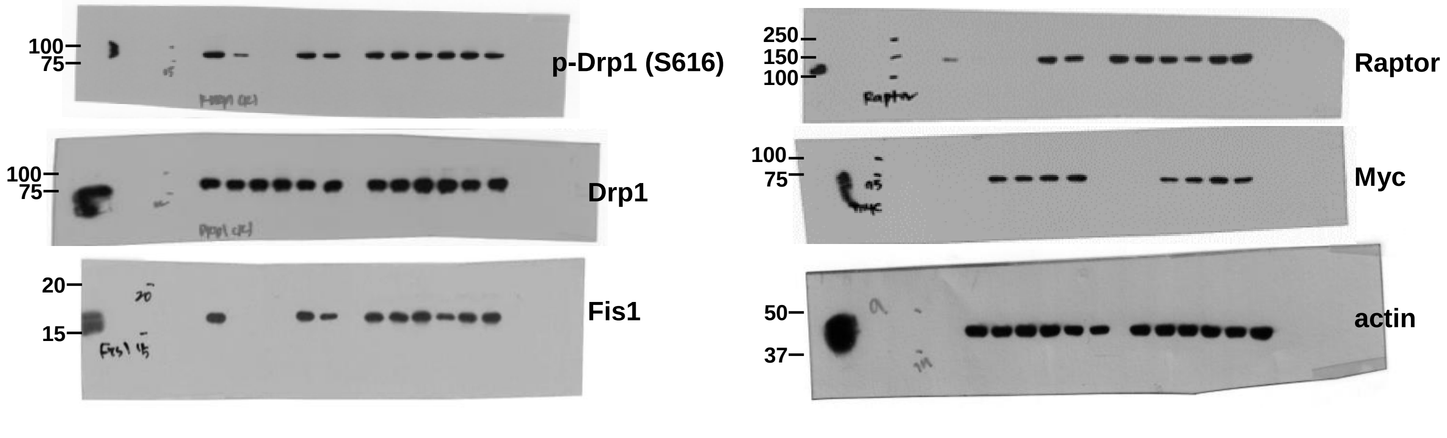

Figure 5A

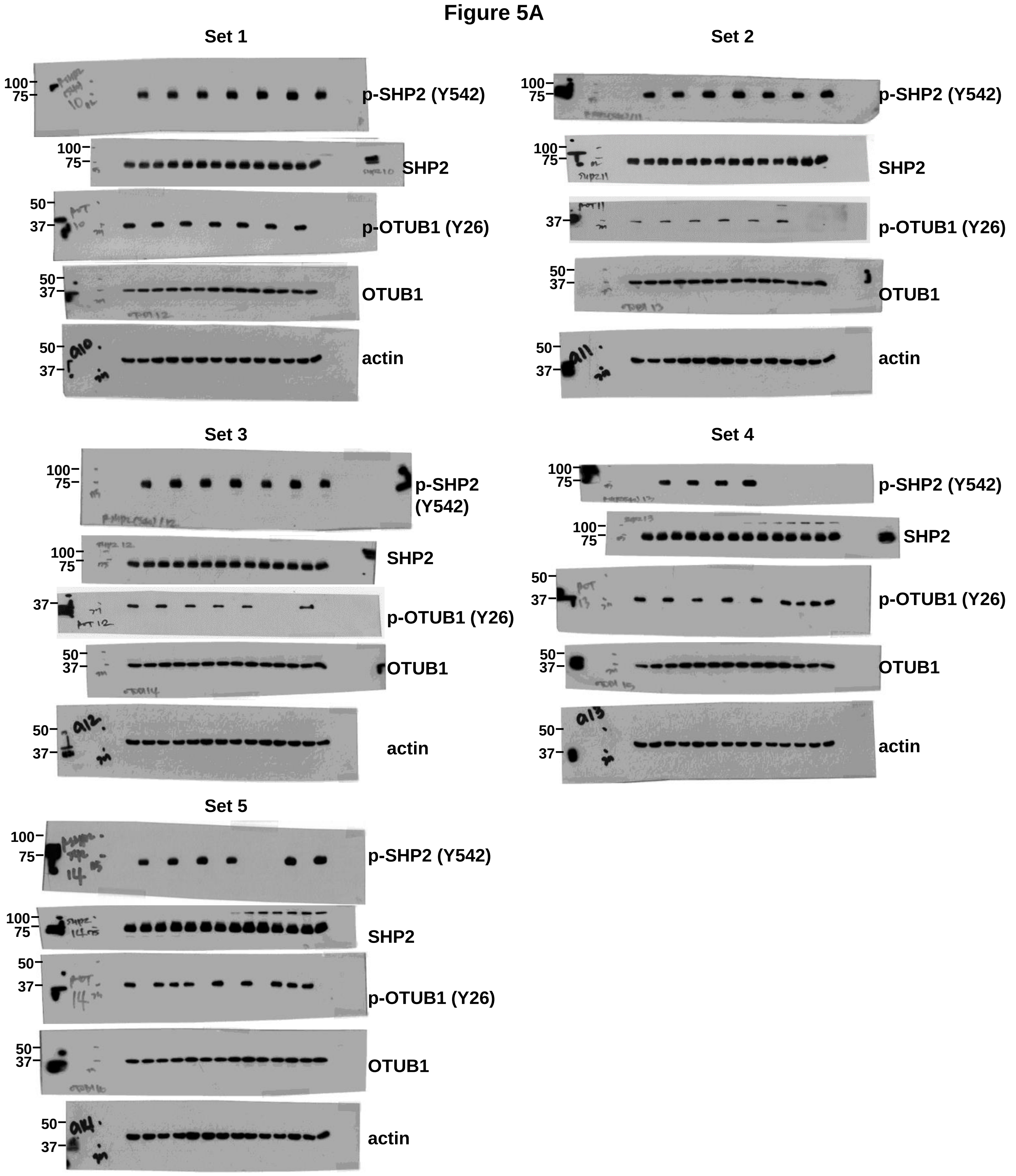

Figure 5B

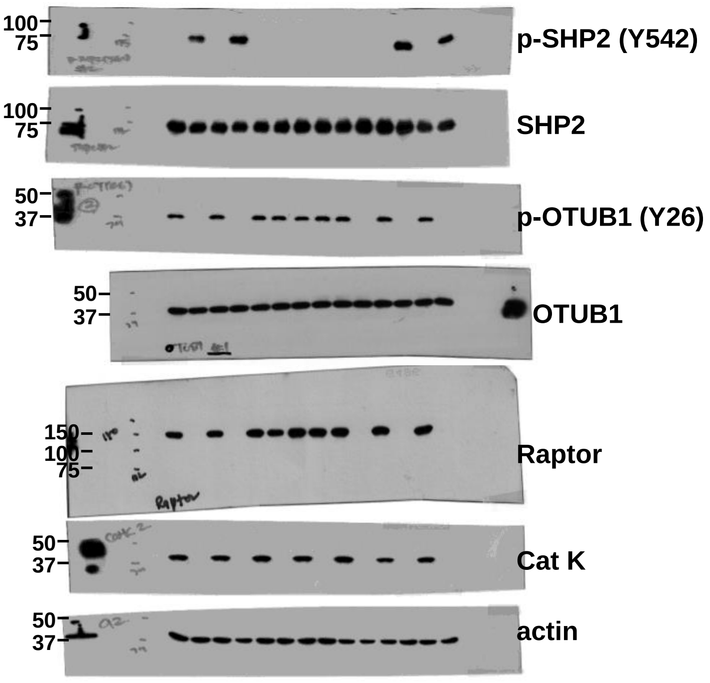

Figure 6A

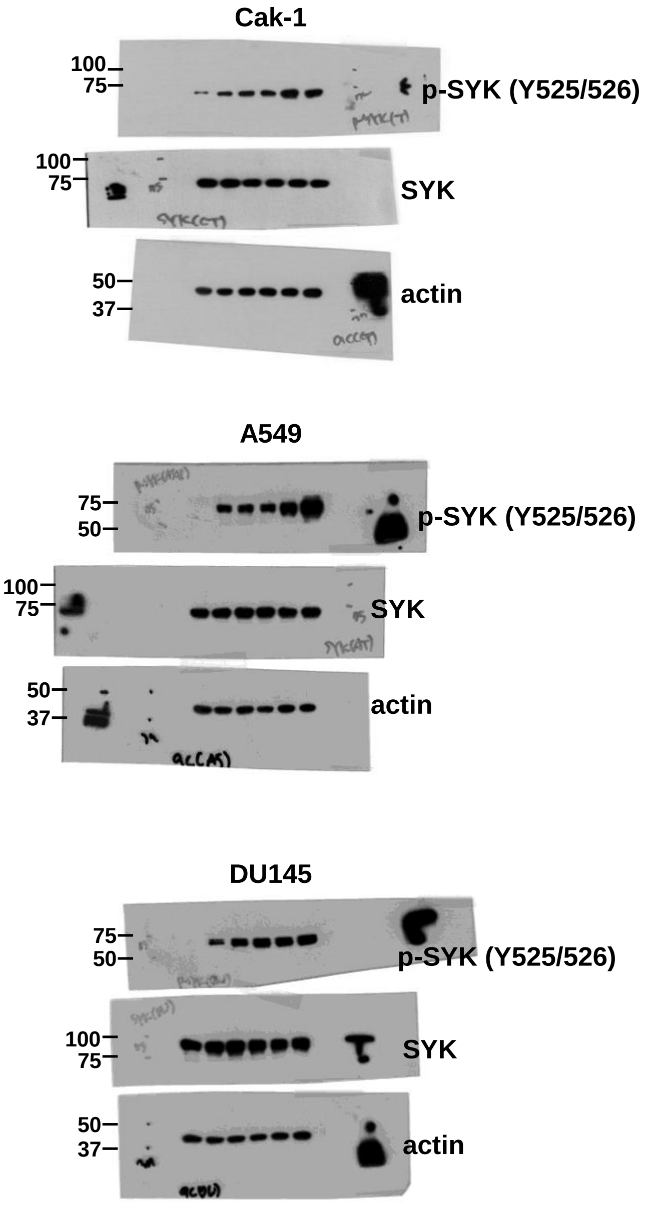

Figure 6B

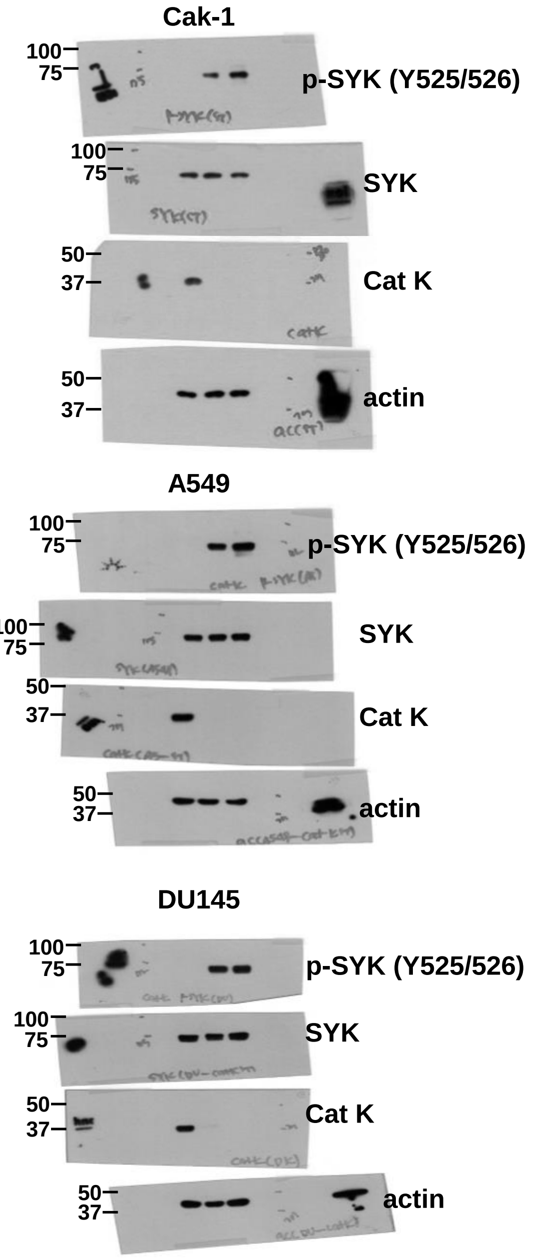

Figure 6C

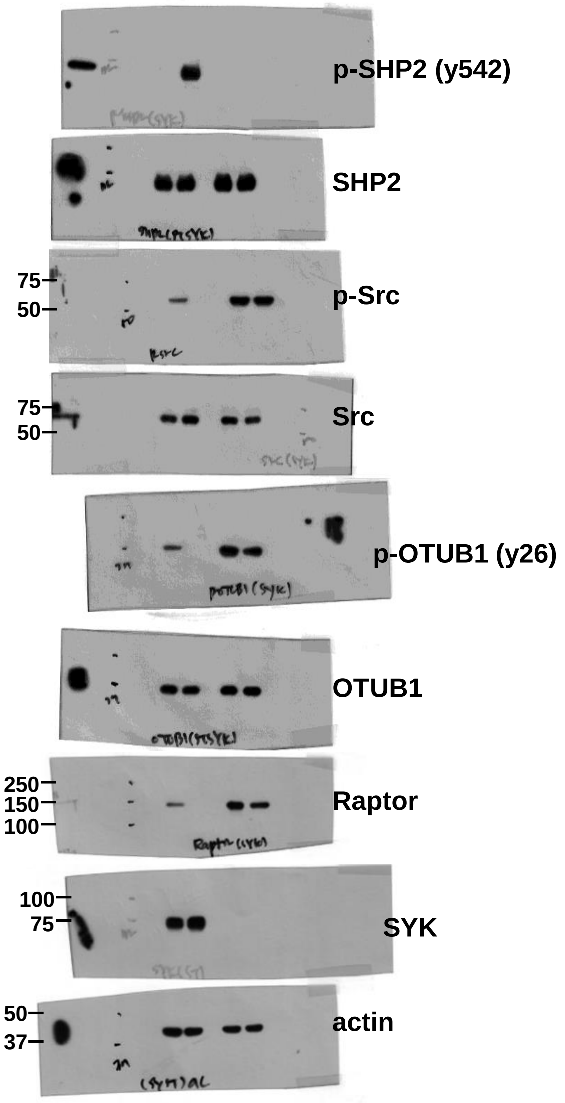

Figure 6D

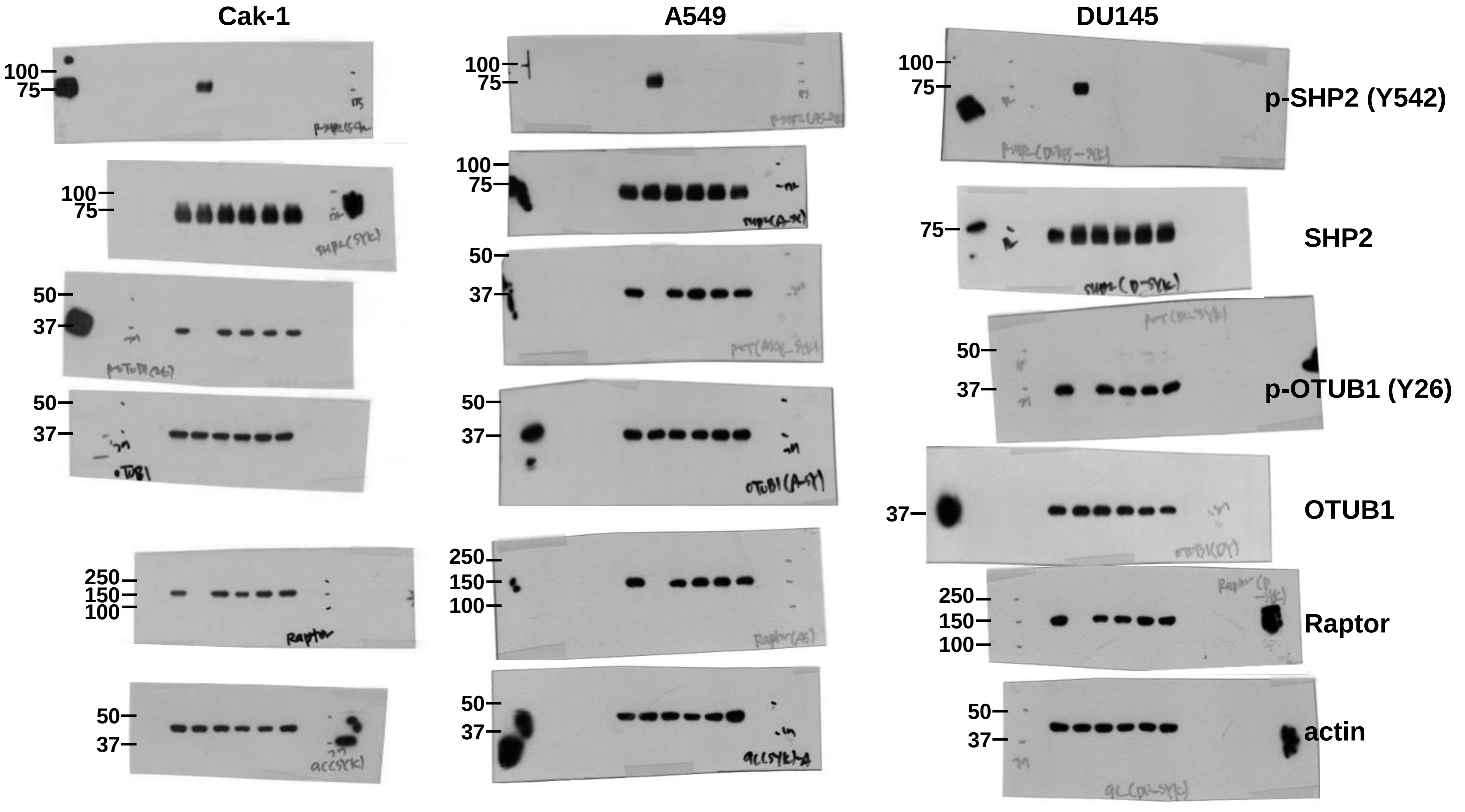

Figure 6I

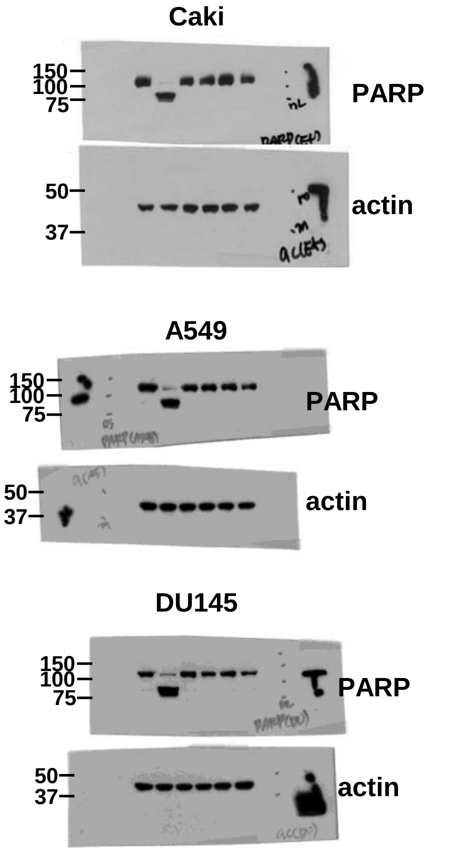

Figure 6J

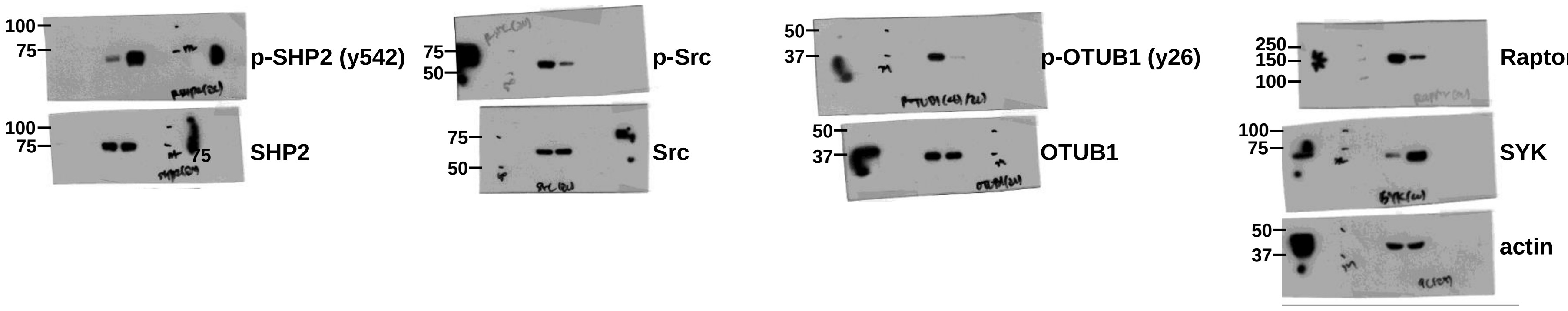

Figure 7A

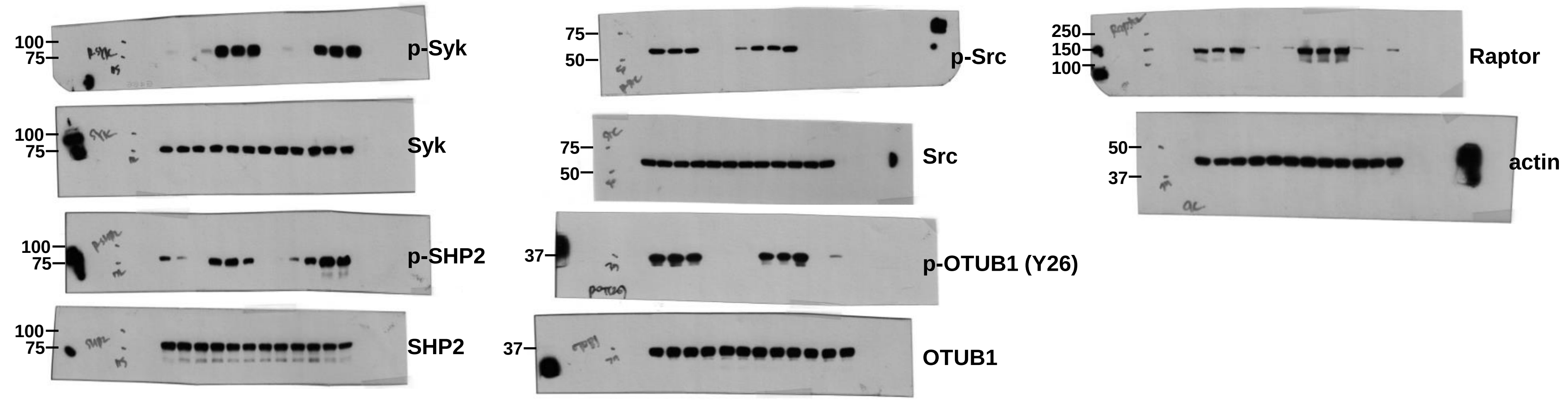

Figure 7C

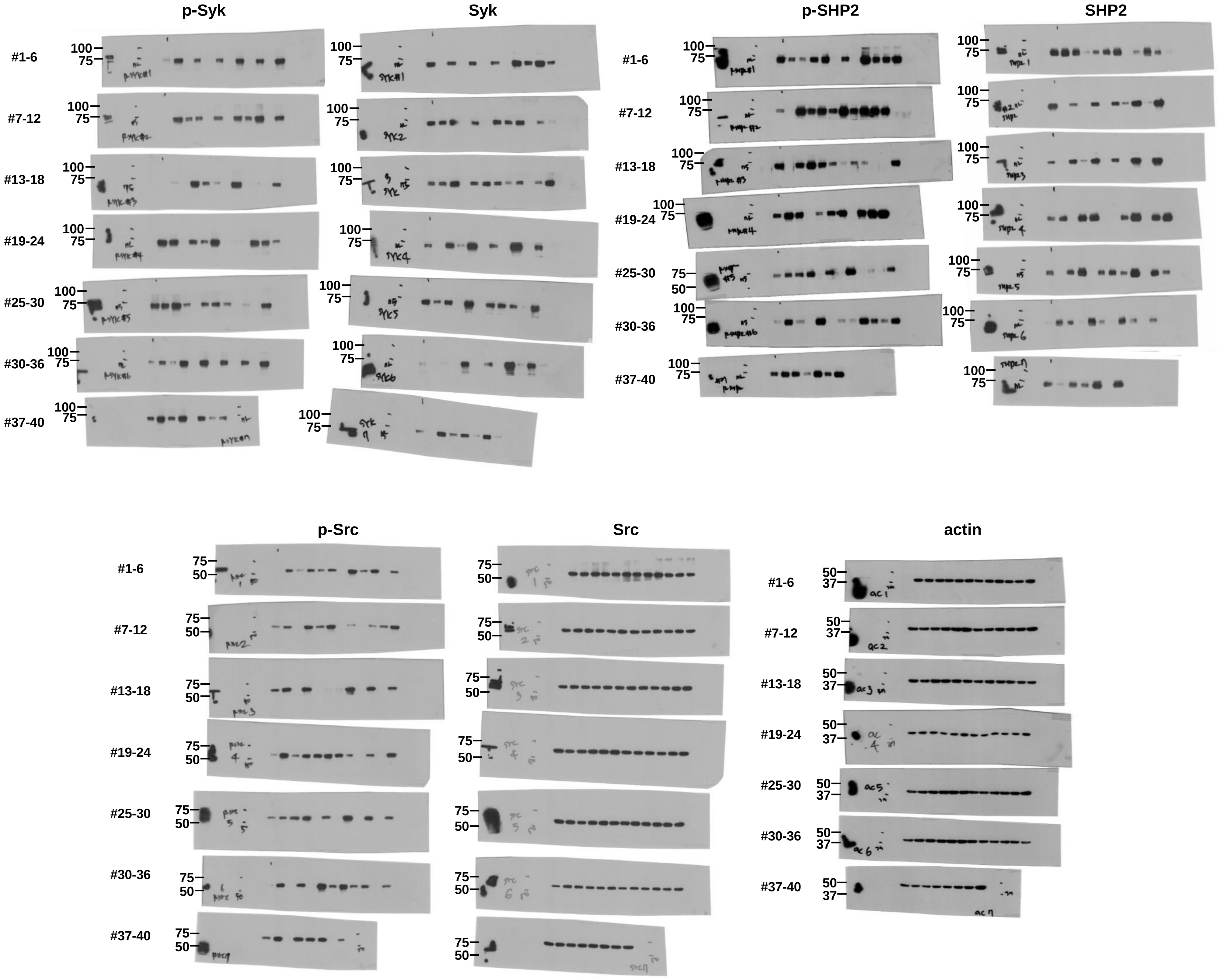

Supplement: Supplementary file 2 — Original Data File [file 41419_2023_5884_MOESM2_ESM.pdf]
