# Supplementary figures and images for: Cathepsin K inhibition induces Raptor destabilization and mitochondrial dysfunction via Syk/SHP2/Src/OTUB1 axis-mediated signaling
Source: Cell Death Dis. 2023 Jun 17;14(6):366. doi: 10.1038/s41419-023-05884-z (PMC10276854; doi:10.1038/s41419-023-05884-z)

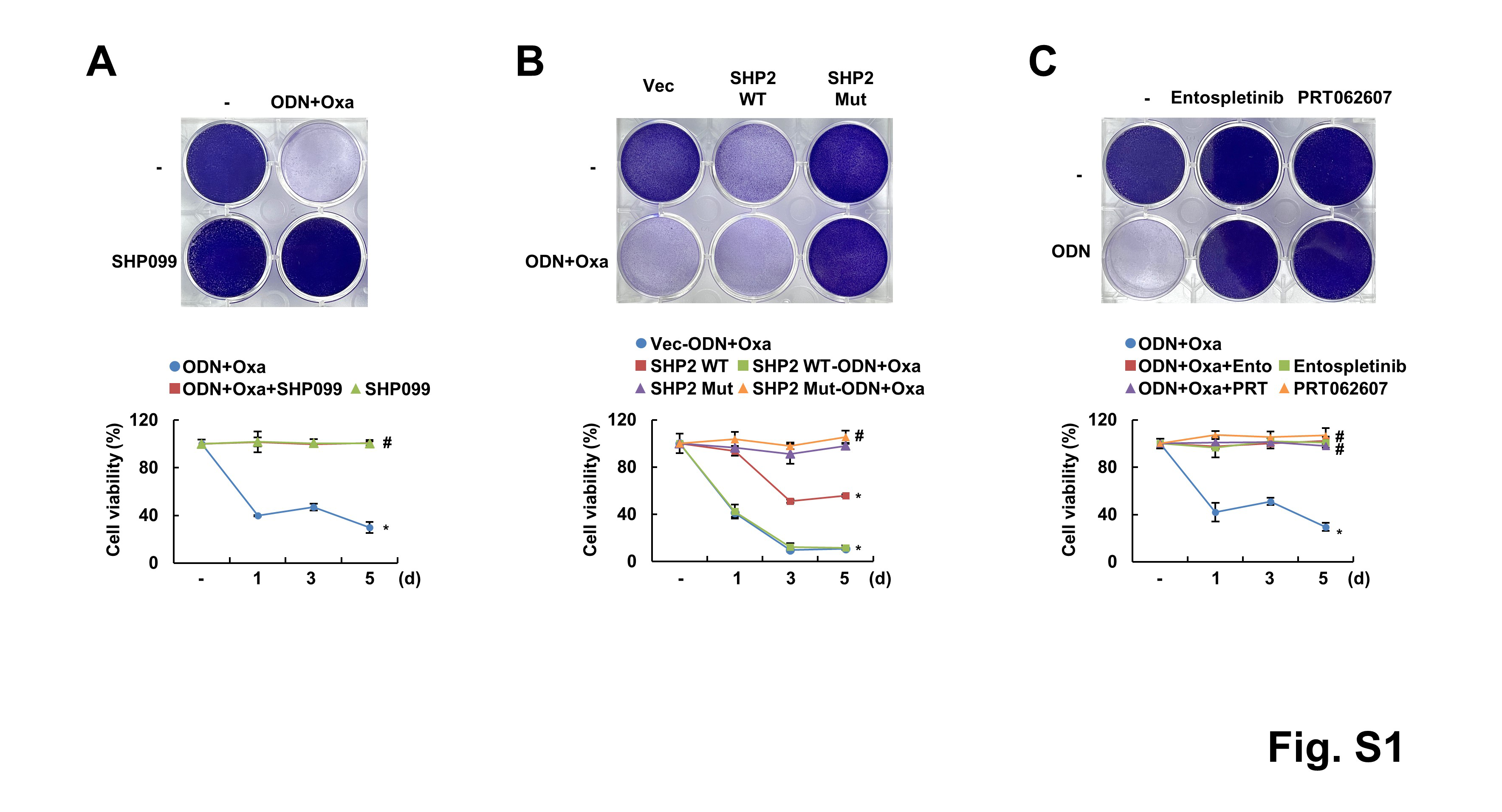

Supplement: Supplementary file 4 — Supplementary Figure S1 [file 41419_2023_5884_MOESM4_ESM.tif]
